# Springing of Atmospheric Pressure Plates While Soldering

**Published:** 1849-07

**Authors:** 


*Springing of Atmospheric Pressure Plates while Soldering.*—Every dentist who has applied many artificial teeth upon this principle, has, doubtless, in some cases, had his plates to warp, while soldering his teeth to them. This has often been a source of much perplexity and inconvenience, and some have occasionally been reduced to the necessity of taking the teeth off, and striking up the plate a second time. For the purpose of remedying this difficulty, Mr. M. W. Sherwood, of Williamstown, Mass., proposes, in the New York Dental Recorder, to "swage the plate after the teeth are soldered on;" and his method of doing this, he thus describes: "I take the model of the jaw, to which I fit the plate, and wetting plaster of paris, rather thick, I build it on to the ridge of the jaw against which the teeth rest, an inch or more high. When the plaster has set, I pare it off smooth, and cast the lead on the die as high as the plaster. When it is cool, I take the plaster out and try the plate. If the teeth touch, I pare off the lead until they do not hit in the least, then putting the die and matrice together, I bring them down with a few smart blows. The most refractory plate is subdued to a perfect fit instantly."

The plan which we have adopted in such cases is much simpler, and almost always proves perfectly successful. It consists in annealing the plate on the plaster model, after having first bound it down closely to it. We would advise trying this plan before resorting to Mr. Sherwood's.

[*Balt. Ed.*

